# An evaluation of the metabolic syndrome in the HyperGEN study

**DOI:** 10.1186/1743-7075-2-2

**Published:** 2005-01-18

**Authors:** Aldi T Kraja, Steven C Hunt, James S Pankow, Richard H Myers, Gerardo Heiss, Cora E Lewis, DC Rao, Michael A Province

**Affiliations:** 1 Division of Biostatistics, Washington University School of Medicine, St. Louis, MO., USA; 2 Department of Medicine, University of Utah School of Medicine, Salt Lake City, UT., USA; 3 Division of Epidemiology, University of Minnesota, Minneapolis, MN., USA; 4 Department of Neurology, Boston Medical Center, MA., USA; 5 Department of Epidemiology, University of North Carolina, Chapell Hill, NC., USA; 6 Division of Preventive Medicine, University of Alabama, Birmingham, AL., USA

## Abstract

**Background:**

In 2001 the National Cholesterol Education Program (NCEP) provided a categorical definition for metabolic syndrome (c-MetS). We studied the extent to which two ethnic groups, Blacks and Whites were affected by c-MetS. The groups were members of the Hypertension Genetic Epidemiology Network (HyperGEN), a part of the Family Blood Pressure Program, supported by the NHLBI. Although the c-MetS definition is of special interest in particular to the clinicians, the quantitative latent traits of the metabolic syndrome (MetS) are also important in order to gain further understanding of its etiology. In this study, quantitative evaluation of the MetS latent traits (q-MetS) was based on the statistical multivariate method factor analysis (FA).

**Results:**

The prevalence of the c-MetS was 34% in Blacks and 39% in Whites. c-MetS showed predominance of obesity, hypertension, and dyslipidemia. Three and four factor domains were identified through FA, classified as "Obesity," "Blood pressure," "Lipids," and "Central obesity." They explained approximately 60% of the variance in the 11 original variables. Two factors classified as "Obesity" and "Central Obesity" overlapped when FA was performed without rotation. All four factors in FA with Varimax rotation were consistent between Blacks and Whites, between genders and also after excluding type 2 diabetes (T2D) participants. Fasting insulin (INS) associated mainly with obesity and lipids factors.

**Conclusions:**

MetS in the HyperGEN study has a compound phenotype with separate domains for obesity, blood pressure, and lipids. Obesity and its relationship to lipids and insulin is clearly the dominant factor in MetS. Linkage analysis on factor scores for components of MetS, in familial studies such as HyperGEN, can assist in understanding the genetic pathways for MetS and their interactions with the environment, as a first step in identifying the underlying pathophysiological causes of this syndrome.

## Background

Metabolic and physiologic disorders for cardiovascular disease (CVD) and type 2 diabetes (T2D), including abdominal obesity, insulin resistance, hyperglycemia, dyslipidemia, and hypertension often cluster. This cluster is frequently identified as the "metabolic syndrome" (MetS). Reaven [1] related MetS to the presence of resistance to insulin-mediated glucose disposal, glucose intolerance, hyperinsulinemia, increased triglycerides, decreased high-density lipoprotein cholesterol, and hypertension. Later, the definition of MetS was extended to include obesity, inflammation, microalbuminuria, and abnormalities of fibrinolysis and of coagulation [2-4]. Clearly, insulin resistance is not considered equivalent to MetS [5,6]. Grundy et al. [7], at a recent National Heart, Lung, and Blood Institute (NHLBI) /American Heart Association (AHA) National Conference, concluded that abdominal obesity is strongly associated with MetS. Sonnenberg et al. [8] have hypothesized that increased adipose tissue mass contributes to the development of MetS by triggering an increase in proinflammatory adipokines, especially the tumor necrosis factor-α, which may play a role in the pathogenesis of dyslipidemia, insulin resistance, hypertension, endothelial dysfunction, and atherogenesis. Although several studies have targeted MetS, its genetic determination and its pathophysiology remain unclear [9].

Different definitions and multivariate statistical approaches have been applied to characterize the increasing high-risk MetS premorbid state. Recently, special attention has received the categorical definition of metabolic syndrome (c-MetS) of the National Cholesterol Education Program Adult Treatment Panel III (NCEP) [2]. The NCEP definition (see Material and Methods) has especially two components, its usefulness in the clinical diagnosis of MetS and its association with recommendations for its therapeutic treatment. Based on the NCEP c-MetS definition, it is reported that 20 to 25 percent of the U.S. adult population has MetS. This represents a high prevalence of the syndrome in the general population [10,11]. In addition, employing the multivariate statistical method factor analysis (FA) different studies in different sampled populations have documented the underlying latent traits of MetS [4,12-17]. Meigs [3] has reported that FA in different studies has yielded on average 2 to 4 latent traits (factors) of MetS. Different studies have found different numbers of latent factors, depending on the type and number of the original risk factors analyzed, sampled population(s), methods utilized, including the statistical rotation method, and decisions about how many factors appeared statistically meaningful.

The objective of this study was to exemplify important facets of the MetS in the HyperGEN study. Two MetS aspects were assessed: **a. **The trait characterized as the categorical MetS (c-MetS) was studied according to the NCEP definition; **b. **The underlying (latent) traits or clusters of MetS (q-MetS) were evaluated by performing FA with and without Varimax rotation on 11 risk factors. All data were grouped by ethnicity and gender. Subgroups were created by excluding T2D participants, under the assumptions that T2D individuals may have a different pattern of glucose and insulin levels. Finally, our goal was to compare the expression of c-MetS and q-MetS in the Hypertension Genetic Epidemiology Network (HyperGEN) study.

## Results

### Sample size and relationships among original risk factors

**Figure 1:**
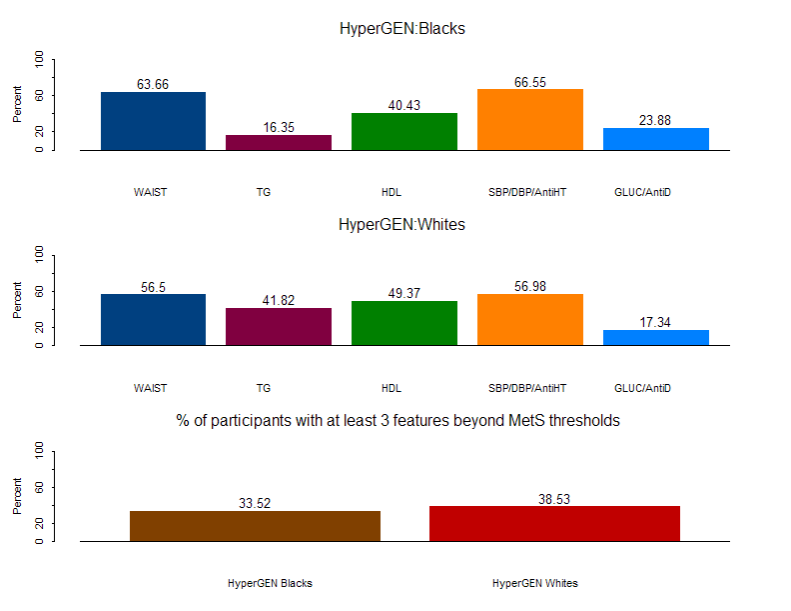
Categorical MetS (c-MetS) in the HyperGEN Study

For c-MetS the sample sizes varied from 2,025 observations for fasting triglycerides (TG) to 2,300 for high density lipoprotein (HDL) cholesterol in Blacks, and from 2,171 observations for TG to 2,471 for HDL in Whites. In the HyperGEN study, a high percentage of individuals have body waist (WAIST) and systolic blood pressure (SBP) / diastolic blood pressure (DBP) above the NCEP thresholds (Figure 1). Whites tended to have greater percentages of participants with TG and HDL beyond the NCEP thresholds than Blacks. The prevalence of c-MetS was 34 and 39 percent in Blacks and Whites, respectively.

For q-MetS, the sample sizes and variables studied are summarized in Table 1 (the statistics were similar when T2D subjects were excluded, results not shown). After participants with missing data for any of the 11 original variables were excluded, this resulted in a sample of 1,422 Blacks and 1,470 Whites with complete data. The samples reduced to 1,173 Blacks and 1,322 Whites when T2D participants were excluded.

**Table 1:** Original Data Included in Factor Analysis

		**Blacks (N = 1422)**				**Whites (N = 1470)**			
		
**Variable**	**Units**	**Kurtosis**	**Skewness**	**Mean**	**Std Dev**	**Kurtosis**	**Skewness**	**Mean**	**Std Dev**
**BMI**	**kg/m**^2^	0.51†	0.72	32.04‡	7.54	0.67	0.77	28.86	5.57
**GLUC**	**mg/dl**	0.68	0.70	107.37	44.06	1.43	-0.55	100.80	26.38
**INS**	**μU/ml**	0.34	0.06	10.55	9.24	0.42	0.23	7.45	5.90
**LDL**	**mg/dl**	0.31	0.33	118.89	36.63	0.36	0.26	116.41	31.56
**HDL**	**mg/dl**	0.46	0.22	53.58	15.17	0.17	0.18	48.70	14.09
**TG**	**mg/dl**	0.41	0.35	101.32	56.03	0.17	0.20	144.77	75.24
**SBP**	**mm Hg**	0.46	0.68	128.45	21.78	0.44	0.62	120.50	18.33
**DBP**	**mm Hg**	0.85	0.60	74.08	11.59	0.42	0.44	68.96	9.94
**WAIST**	**cm**	0.51	0.64	102.05	17.78	0.74	0.71	99.22	15.48
**WHR**	**ratio**	0.11	0.10	0.90	0.08	0.17	0.05	0.92	0.09
**%BF**	**%**	0.44	0.17	40.00	12.06	0.33	0.57	33.41	9.29

†Kurtosis and skewness are reported after the data were transformed (where necessary) and adjusted (see Material and Methods); ‡number of observations, mean, and standard deviations represent measures from the final sample in factor analysis

In terms of the participants with complete data, the age at clinic visit had a mean of 46 and a standard deviation of 13 years in Blacks, and a mean of 51 and a standard deviation of 14 years in Whites. Overall, when compared to Whites, Blacks tended to have a higher body mass index (BMI), fasting plasma glucose (GLUC), fasting insulin (INS), HDL, SBP, DBP, WAIST, and percent body fat (%BF), similar low density lipoprotein (LDL) cholesterol and waist to hip ratio (WHR), and lower TG. Kurtosis after adjustments varied from 0.11 for WHR to 0.85 for DBP in Blacks, and 0.17 for WHR to 1.43 for GLUC in Whites, which demonstrates normal distributions for the traits in study and also for the factors created by performing FA (see Material and Methods).

Pearson correlations among the 11 adjusted and normally distributed variables are presented in Table 2. The lower triangle correlations correspond to all data, whereas the upper triangle refers to the data excluding T2D participants. In both ethnicities, strong correlations were observed among BMI, WAIST, %BF, INS, and WHR. GLUC correlated significantly with INS (inversely, because GLUC was inversely squared transformed). HDL cholesterol had a significantly negative correlation with TG and INS. SBP was significantly correlated with DBP. Interestingly, WAIST correlated higher with BMI (about 0.9) than with WHR (about 0.7) in all groups. These inter-correlations determine the structure of the factors.

**Table 2:** Correlation Matrix of the Variables Included in Factor Analysis

	Variables **	Blacks: Upper Triangle: All Data Excluding T2D										
	(N = 1422 / 1173)	BMI	INS	GLUC	LDL	HDL	TG	SBP	DBP	WAIST	WHR	%BF
Lower Triangle:All Data	BMI		**0.56‡**	**- 0.33‡**	**0.15‡**	**- 0.22‡**	**0.15‡**	**0.18‡**	**- 0.04**	**0.91‡**	**0.45‡**	**0.78‡**
	INS	**0.51‡**		**- 0.50‡**	**0.16‡**	**- 0.37‡**	**0.32‡**	**0.04**	**- 0.08†**	**0.57‡**	**0.44‡**	**0.49‡**
	GLUC	**- 0.29‡**	**- 0.40‡**		**- 0.16‡**	**0.25‡**	**- 0.26‡**	**- 0.06**	**0.03**	**- 0.33‡**	**- 0.26‡**	**- 0.28‡**
	LDL	**0.13‡**	**0.11‡**	**- 0.11‡**		**- 0.18‡**	**0.23‡**	**- 0.00**	**- 0.05**	**0.14‡**	**0.11†**	**0.17‡**
	HDL	**- 0.20‡**	**- 0.35‡**	**0.23‡**	**- 0.16‡**		**- 0.41‡**	**0.02**	**0.06**	**- 0.25‡**	**- 0.25‡**	**- 0.20‡**
	TG	**0.17‡**	**0.32‡**	**- 0.31‡**	**0.20‡**	**- 0.41‡**		**0.05**	**- 0.01**	**0.20‡**	**0.28‡**	**0.16‡**
	SBP	**0.17‡**	**0.02**	- 0.07*	**0.02**	**0.05**	**0.06**		**0.76‡**	**0.16‡**	**0.12†**	**0.06**
	DBP	**- 0.04**	**- 0.08†**	**0.04**	**- 0.02**	**0.08†**	**- 0.00**	**0.74‡**		**- 0.03**	**0.03**	**- 0.06**
	WAIST	**0.90‡**	**0.52‡**	**- 0.32‡**	**0.14‡**	**- 0.23‡**	**0.22‡**	**0.15‡**	**- 0.03**		**0.70‡**	**0.75‡**
	WHR	**0.44‡**	**0.42‡**	**- 0.32‡**	**0.10†**	**- 0.25‡**	**0.30‡**	**0.11‡**	**0.02**	**0.69‡**		**0.407‡**
	%BF	**0.76‡**	**0.44‡**	**- 0.24‡**	**0.16‡**	**- 0.18‡**	**0.16‡**	**0.03**	- 0.06*	**0.74‡**	**0.37‡**	

	Variables	Whites: Upper Triangle: All Data Excluding T2D										
	(N = 1470 / 1322)	BMI	INS	GLUC	LDL	HDL	TG	SBP	DBP	WAIST	WHR	%BF

Lower Triangle:All Data	BMI		**0.55‡**	**- 0.30‡**	**0.01**	**- 0.21‡**	**0.20‡**	**0.19‡**	0.07*	**0.89‡**	**0.46‡**	**0.77‡**
	INS	**0.52‡**		**- 0.30‡**	**- 0.01**	**- 0.35‡**	**0.34‡**	**0.22‡**	**0.15‡**	**0.52‡**	**0.36‡**	**0.45‡**
	GLUC	**- 0.32‡**	**- 0.31‡**		- 0.08*	**0.13‡**	**- 0.16‡**	**- 0.12†**	- 0.09*	**- 0.30‡**	**- 0.25‡**	**- 0.26‡**
	LDL	**0.02**	**- 0.02**	**- 0.05**		**- 0.04**	0.08*	**0.05**	**0.07**	**0.04**	0.08*	0.07*
	HDL	**- 0.22‡**	**- 0.37‡**	**0.21‡**	**- 0.02**		**- 0.43‡**	**0**	**0.01**	**- 0.20‡**	**- 0.21‡**	**- 0.12†**
	TG	**0.20‡**	**0.33‡**	**- 0.21‡**	0.06*	**- 0.45‡**		**0.13†**	**0.09†**	**0.22‡**	**0.23‡**	**0.20‡**
	SBP	**0.17‡**	**0.19‡**	**- 0.13‡**	**0.04**	**0.00**	**0.10†**		**0.70‡**	**0.15‡**	**0.13‡**	**0.09†**
	DBP	**0.03**	**0.09†**	**0.00**	**0.06**	**0.01**	**0.06**	**0.67‡**		**0.06**	**0.09†**	**0.03**
	WAIST	**0.89‡**	**0.49‡**	**- 0.33‡**	**0.04**	**- 0.21‡**	**0.23‡**	**0.14‡**	0.02*		**0.70‡**	**0.77‡**
	WHR	**0.46‡**	**0.35‡**	**- 0.25‡**	**0.06**	**- 0.23‡**	**0.25‡**	**0.11†**	0.07*	**0.69‡**		**0.44‡**
	%BF	**0.76‡**	**0.43‡**	**- 0.25‡**	**0.08†**	**- 0.12‡**	**0.18‡**	**0.11†**	**0.01**	**0.76‡**	**0.43‡**	

*p < 0.05 † < 0.01 ‡ < 0.0001

**Variables were adjusted for age and center within ethnicity and gender (see Material and Methods)

A negative correlation between GLUC and INS is result of the inverse squared transformation of the original GLUC

FA with no rotation identified a factor (Factor 1) that was loaded mostly by central obesity, obesity risk factors and INS (Table 4, see Additional file 1 ). It explained 21 percent to 32 percent of the variance of the original risk factors. Three other factors, "Obesity," "Blood pressure," and "Lipids," were identified. In Blacks, the "Obesity" factor was primarily loaded by BMI, INS, WAIST, and %BF. In Whites, exclusion of T2D participants led to a similar pattern. However, in all data in Whites, the first factor represented a stronger mixture of central obesity, obesity and INS, leaving the fourth factor with mainly WHR loading. In order to distinguish the second factor from the first one, we labeled the first as "Central obesity" factor and the second as "Obesity" factor. In both ethnicities, blood pressure (BP) gave rise to a separate factor. Also the "Lipids" remained as a separate factor and was dominated mainly by HDL and TG. INS was associated mostly with the "Obesity" and "Lipids" factors.

In the case of FA with Varimax rotation, again, 4 distinct factors explained about 60 percent of the variance in the original variables (Table 4, see Additional file 1). We are labeling them as "Obesity," "BP," "Lipids," and "Central obesity". Factor loadings less than 0.1 were not listed in Tables 3 and 4 (see Additional file 1). The first factor alone explained 23 percent to 25 percent of the variance in the original risk factors, while the fourth factor explained 7 percent to 9 percent of the variance. The "Obesity" factor (Factor 1) loaded mainly BMI, WAIST, WHR, %BF, and INS. SBP and DBP loaded separately. A distinct factor ("Lipids") loaded mainly HDL cholesterol, TG, INS and GLUC in Blacks and HDL cholesterol, TG and INS in Whites. The fourth factor contained a higher loading for WHR than for WAIST. Similar factor loadings were present in the samples when T2D participants were excluded (Table 4, see Additional file 1).

**Table 3:** Factors, Loadings, and Sums of Squared Loadings in All Data (Males (M) and Females (F)), and by Gender (M, F) (Varimax Rotation)

**Factor 1 (Obesity)**	Sample	BMI*	**%BF**	**WHR**	**WAIST**	**INS**	**GLUC**	**SBP**	**DBP**	**LDL**	**HDL**	**TG**	**SS Loadings**
Blacks	**M+F (1422)**	**0.95†**	**0.71**	0.32	**0.86**	**0.42**	-0.20	0.13					2.49
	**M (483)**	**0.92**	**0.71**	**0.65**	**0.95**	**0.46**	-0.15	0.11		0.11	-0.21	0.14	2.91
	**F (939)**	**0.96**	**0.71**	0.23	**0.84**	**0.40**	-0.22	0.14					2.43
													
Whites	**M+F (1470)**	**0.94**	**0.77**	**0.40**	**0.86**	**0.46**	-0.26	0.14			-0.11	0.11	2.69
	**M (721)**	**0.94**	**0.76**	**0.55**	**0.93**	**0.48**	-0.26				-0.13	0.16	2.99
	**F (749)**	**0.94**	**0.81**	0.31	**0.84**	**0.46**	-0.28	0.18			-0.11	0.11	2.69

**Factor 2 (BP)**	Sample	**BMI**	**%BF**	**WHR**	**WAIST**	**INS**	**GLUC**	**SBP**	**DBP**	**LDL**	**HDL**	**TG**	**SS Loadings**

Blacks	**M+F (1422)**							**0.99**	**0.76**				1.58
	**M (483)**					0.13		**0.83**	**0.83**				1.42
	**F (939)**							**0.99**	**0.77**				1.59
													
Whites	**M+F (1470)**					0.13		**0.88**	**0.76**				1.39
	**M (721)**					0.14		**0.87**	**0.80**				1.41
	**F (749)**					0.12	-0.14	**0.98**	**0.65**		0.11		1.45

**Factor 3 (Lipids)**	Sample	**BMI**	**%BF**	**WHR**	**WAIST**	**INS**	**GLUC**	**SBP**	**DBP**	**LDL**	**HDL**	**TG**	**SS Loadings**

Blacks	**M+F (1422)**	0.22	0.19	0.31	0.24	**0.52**	**-0.43**			0.27	**-0.57**	**0.64**	1.49
	**M (483)**	0.17	0.12	0.29	0.18	**0.48**	-0.19				**-0.72**	**0.65**	1.37
	**F (939)**	0.18	0.11	0.29	0.21	**0.49**	**-0.41**			0.32	**-0.55**	**0.59**	1.32
													
Whites	**M+F (1470)**	0.23	0.17	0.27	0.21	**0.51**	-0.27				**-0.68**	**0.64**	1.41
	**M (721)**	0.14				**0.41**	-0.18			-0.18	**-0.75**	**0.61**	1.20
	**F (749)**	0.25	0.19	0.24	0.23	**0.5**	-0.34	0.11		0.22	**-0.70**	**0.60**	1.44

**Factor 4 (Central Obesity)**	Sample	**BMI**	**%BF**	**WHR**	**WAIST**	**INS**	**GLUC**	**SBP**	**DBP**	**LDL**	**HDL**	**TG**	**SS Loadings**

Blacks	**M+F (1422)**			**0.90**	**0.40**	0.15	-0.13					0.11	1.03
	**M (483)**		-0.19	0.37	0.11								0.2
	**F (939)**			**0.93**	**0.44**	0.15	-0.16					0.11	1.13
													
Whites	**M+F (1470)**		0.16	**0.69**	**0.47**		-0.10						0.76
	**M (721)**		0.11	**0.57**	0.27		-0.11				0.13		0.45
	**F (749)**			**0.92**	**0.44**	0.14							1.10

* Variables were adjusted for age and center within ethnicity and gender (see Material and Methods)

GLUC negative loadings are result of inverse squared power transformation of the original GLUC;† Loadings ≥ 0.4 are in bold

FA with Varimax rotation was performed also by gender (Table 3). Between genders, the factors loaded in a similar fashion in Blacks and Whites. WHR and WAIST reflected gender differences in their loadings in factors 1 and 4.

## Discussion

The fact that MetS is more prevalent in the HyperGEN study as compared to the average of the US adult population [10,11], is probably due to selection bias arising from the hypertension selection criterion applied in HyperGEN (see Material and Methods). Although BP was an important contributor to the categorical definition of the MetS (c-MetS), other risk factors such as WAIST, HDL and TG were also important. The HyperGEN Whites had a higher prevalence of c-MetS than the Blacks. Blacks had a higher percentage of participants with WAIST, BP, and GLUC beyond the NCEP thresholds.

In this study, FA of 11 potential risk factors for CVD and T2D yielded 4 latent factors, explaining about 60 percent of the variance in the original risk factors. Using the maximum likelihood estimation (MLE) method in S-PLUS (Insightful Corporation software), we found that the model p-values were significant, suggesting that additional factors may exist. However, although additional factors must exist to explain approximately 40 percent of the variance in the original variables, none of the remaining factors individually can explain more than about 5 percent of the variance. Therefore, we concluded that the quantitative structure of MetS can be described in terms of three to four factors when no rotation was performed, and four factors with Varimax rotation. One may even argue whether the fourth factor in the Varimax rotation is very meaningful. We chose to retain the "Central Obesity" factor particularly because it tends to reflect the well-known gender asymmetry (Table 3). FA without and with Varimax rotation can be useful in different settings. We believe that FA without any rotation makes more sense when investigating the pattern of risk factor clustering in the MetS. On the other hand, gene finding studies can be enhanced with Varimax rotation because, it is easier to find genes each of which influences a different (uncorrelated) factor. Therefore, depending on the goals of a study, rotation may or may not be used. We regard this flexibility as strength of the FA method.

We tested the pattern of the factors between genders only for FA with Varimax rotation. This pattern was stable among ethnicities between genders (Table 3). WHR on Factors 1 and 4 and WAIST on Factor 4 had statistically different loadings in males and females.

Another characteristic of the HyperGEN study was that at least two participants in each sibship had hypertension. A large proportion of the hypertensive participants have used anti-hypertensive and anti-cholesterol medications. Maison et al. [13] have compared medicated and unmedicated groups to see any implications of the medication use in FA. They applied FA to 9 risk factor changes over time, and separated data into groups of treated and untreated for hypertension and dyslipidemia. They found 3 and 4 factors respectively in males and females, the "BP", "Glucose," "Lipid," and "BMI, WHR, INS and TG" factors, which were similar between treated and untreated groups.

It is a common belief that T2D participants may have a different expression of INS and GLUC, therefore it may influence also the factors pattern in the MetS analysis. In the present study, we found a consistency of the factors before and after removing type 2 diabetics. This finding is supported also by Hanson et al. [17] who studied two samples of Pima Indians classified as T2D and non-T2D. They identified consistently 4 latent factors out of 10 risk factors in the two samples, with a relative variation only on insulin loadings. They found that the "Insulinemia" factor explained 30 percent of the original variance, "Body size" 20 percent, "BP" 15 percent, and "Lipids" 14 percent. In our study, the INS variable loaded with BMI, WAIST, %BF, and also with lipids. INS was present mainly in 2 and sometimes in 3 factors with loadings mostly in the "Obesity" and "Lipids" factors.

Other studies have provided similar results about the latent traits of MetS [4,16,17]. FA studies cited here, and other studies described by Meigs [3], have elements in common with our study: 2 to 4 factors were identified; BMI, INS, WHR and WAIST are major contributing risk factors; SBP and DBP load in a separate factor; INS was associated with more than one factor and mainly with obesity and lipids.

Our study and several others have shown that FA is a useful method for studying the underlying traits of MetS. Nevertheless this methodology has not passed without been criticized. Lawlor et al. [18] reviewed 22 published studies of the MetS, all based on FA. None of the studies had clearly identified whether they used FA for exploratory analysis or for hypothesis testing purposes. Such ambiguous use was regarded by Lawlor et al [18] as a major misuse of FA in the study of the metabolic syndrome. In fact exploratory FA and the hypothesis-testing (confirmatory) FA are two distinct methods. Basically, the two analyses use different constraints and different approaches in the analytical software. The exploratory FA is driven by the data (example is our study). On the other hand, the hypothesis-testing FA is only performed with some prior knowledge of possible loadings for different risk factors. One applies confirmatory FA on the data to test if the factor structure of the hypothesized model specifying the interrelationships among the original variables and the latent factors included in the model is true or not. A detailed example of the confirmatory FA of the metabolic syndrome is provided by Shen et al. [16].

If c-MetS and q-MetS are explaining the same disorder in two different aspects in the HyperGEN study, can FA contribute to MetS gene finding? MetS is recognized as a precursor for cardiovascular disease and type 2 diabetes [19]. There are several studies that have used FA for understanding the complexity of the MetS. Our study brings in more evidence that FA provides not only insights about the latent factor traits for MetS, but it produces factor scores for each of the MetS domains at the same time. Can factor scores be used in genetic analysis, such as in linkage analysis? The concept of a latent factor has much (intellectual) parallel with the concept of a latent gene. Much like a latent gene might have pleiotropic effects on several correlated phenotypes (original risk factors), several correlated risk factors load onto a latent factor. This makes FA very attractive from a genetic analysis point of view since, unlike individual risk factors each of which may entail several genes, each factor is likely to involve only a few genes which simplify their discovery. We believe that FA is useful for complex disease gene finding. In a near future motivated from this analysis we plan in the HyperGEN study to perform linkage analysis on the trait established by c-MetS and also on factor scores created by q-MetS, for identifying essential MetS putative genes / QTLs.

## Conclusions

These analyses demonstrated that obesity and hypertension were the most important factors contributing to the MetS in the HyperGEN Study. Three to four distinct factor domains were identified depending on the FA rotation applied and decisions made. Results support the hypothesis that MetS is a compound phenotype, where obesity and its relationship to lipids and insulin are clearly the driving force of MetS. Insulin may play a connecting role between obesity and lipid domains.

In genetic analysis, it is well known that categorical data, especially a complex trait such as MetS, encounter reduced power as compared to quantitative variables. Therefore, we suggest that genetic analysis should be performed on specific combinations of traits that belong to a factor. It is possible that some common genes may exist in the pathways for the factors identified. Linkage analysis investigating putative quantitative trait loci for MetS factor domains can be a first step which may help discover the underlying mechanisms, or generate new hypotheses, in finding the causes of MetS.

## Material And Methods

### Data collection and MetS definition

The sample represents data from the HyperGEN network, part of the Family Blood Pressure Program, supported by the NHLBI as described by Williams et al. [20] and Province et al. [21]. The ethnicity was recorded as a self-reported demographic category. In the HyperGEN study sibships were recruited, each with at least 2 members who were hypertensive before age 60. Also, parents and offspring of some of the hypertensive sibs, as well as random samples of unrelated Blacks and Whites, were recruited, totaling 4,781 participants. Insulin measurements are not available in a part of the sample and therefore the sample size was smaller for FA. Also, participants with missing values for any of the quantitative risk factors used in the definition of MetS were excluded. A detailed account is provided in the Results section.

A participant was classified as having T2D if (s)he had a fasting plasma glucose value ≥ 126 mg/dl, or is a current user of hypoglycemic medication or insulin that was documented at examination in the clinic, or if diabetes was reported in the HyperGEN questionnaire. Also, an age at diagnosis ≥ 40 years was required for T2D individuals [22].

c-MetS according to the NCEP definition, was identified in participants by the simultaneous presence of 3 or more of the following conditions: WAIST > 102 cm in men, and > 88 cm in women; TG ≥ 150 mg/dl; high density lipoprotein (HDL) < 40 mg/dl in men, and < 50 mg/dl in women; systolic blood pressure (SBP) ≥ 130 mm Hg and/or diastolic blood pressure (DBP) ≥ 85 mm Hg or using antihypertensive medications; GLUC ≥ 110 mg/dl or on treatment for diabetes [2].

Factor analysis was founded on 11 variables: BMI expressed as the ratio of body weight divided by body height squared (in kg/m^2^); WAIST measured at the level of the umbilicus in cm; WHR defined as waist circumference divided by hip circumference; GLUC in mg/dl; INS in μU/ml (where fasting time was defined as ≥ 12 hours before blood draw); LDL in mg/dl; HDL in mg/dl; TG in mg/dl; Sitting SBP in mm Hg; DBP in mm Hg (SBP and DBP were measured three times after the subject was asked to sit for five minutes, with the mean of the second and third measurements of each variable being used in the analysis); %BF derived from the bioelectric impedance measurements based on the Lukaski formula [23].

### Statistical Analysis

TG and INS had skewed distributions. A relatively skewed distribution was also present for HDL. Log transformation brought these variables distributions to approximately normal. GLUC and %BF were highly kurtotic. Using Box-Cox transformation, it was found that the inverse of the squared transformation of GLUC (1/GLUC^2^) and the squared transformation of %BF (%BF^2^) reduced the excess kurtosis considerably. The procedure *transreg *in SAS (version 9 for PC) was employed for finding power transformations.

There were two field centers recruiting Blacks and four field centers recruiting Whites. Accordingly, dummy (0,1) field center variables, one for Blacks and three for Whites, were created. All 11 risk factors were adjusted within ethnicity and gender for age, age^2^, age^3^, and field center effects using stepwise regression analysis within ethnicity and gender by employing SAS (SAS version 8.2 for Linux). Any variables with outliers beyond ± 4 standard deviations (SD) were also adjusted for heteroscedasticity of the variance. After the adjustments for each variable, outliers beyond ± 4 SD were eliminated. Each final adjusted variable was standardized to a mean 0 and variance 1.

Prevalence of c-MetS was estimated with the FREQ procedure of SAS. The multivariate method of factor analysis was employed for reducing a group of risk factors to a subset of latent factors. The primary goal of FA is to identify the interrelationships among a set of variables. In this study FA was used for exploratory analysis, because there was no *a priori *information about the structure underlying the variables. FA can be used also for a confirmatory analysis, when validation (or refutation) of a postulated structure is sought. In either case, FA seeks parsimony by summarizing a large group of interrelated variables (risk factors for a complex disease such as MetS) in terms of a small number of latent factors, thereby reducing the dimensionality. Theoretical statistical descriptions of FA can be found in the literature [24-26]. FA was performed with S-PLUS 6.0.1 software by using the *factanal *function, in which the MLE was employed. FA evaluated latent factors underlying the 11 original variables.

FA was performed with and without the Varimax rotation. "No rotation" achieves the simplest latent factor structure, in the extreme case loading any variable in one of the factors and almost negligible loadings in the rest of the factors. That is the reason why some studies (extracting factors with no rotation) find a concentration of the major variables' loading on the first factor. This is also the reason why some investigators named the first factor in their studies as the "Metabolic syndrome" factor [3,27]. Conversely, when Varimax rotation is applied, the objective is to maximize the independence of the clusters for variables that load onto factors. This is achieved by loading in separate factors distinct combinations of the interrelated risk factors. A loading of 0.4 or larger was considered as a significant contribution of an original variable to a factor.

## List of abbreviations used

MetS, metabolic syndrome; c-MetS, categorical MetS; q-MetS, latent traits of MetS; FA, Factor analysis; MLE, maximum likelihood estimate; T2D, type 2 diabetes; CVD, cardiovascular disease; BMI, body mass index; INS, fasting insulin; GLUC, fasting glucose; WHR, waist to hip ratio; SBP, systolic blood pressure; DBP, diastolic blood pressure; BP, blood pressure; TG, fasting triglycerides; LDL, low density lipoprotein cholesterol; HDL, high density lipoprotein cholesterol; %BF, percent body fat.

## Competing interests

The author(s) declare that they have no competing interests.

## Authors' contributions

All authors were equally involved in designing the MetS study, evaluating statistics, interpreting the data, writing the manuscript, and organizing the figure and tables.

## Supplementary Material

Additional File 1 Table 4. This table contains information on factor loadings result of FA with and without rotation performed on 11 risk factors Click here for file
