# Supplementary material for: An evaluation of the metabolic syndrome in the HyperGEN study
Source: Nutr Metab (Lond). 2005 Jan 18;2:2. doi: 10.1186/1743-7075-2-2 (PMC549210; doi:10.1186/1743-7075-2-2)
Supplement: Additional File 1 — Table 4. This table contains information on factor loadings result of FA with and without rotation performed on 11 risk factors [file 1743-7075-2-2-S1.pdf]

Table 4. Factor Loadings Result of FA with and without Rotation Performed on 11 Risk Factors

Rotation="None"	Blacks: All Data				Blacks: Excluding T2D			
	Factor 1	Factor 2	Factor 3	Factor 4	Factor 1	Factor 2	Factor 3	Factor 4
Proportion of Variance	**0.21**	**0.17**	**0.14**	**0.08**	**0.21**	**0.17**	**0.15**	**0.08**
Cumulative Variance	**0.21**	**0.38**	**0.52**	**0.60**	**0.21**	**0.38**	**0.52**	**0.60**
Loadings: BMI†	**0.45†**	**0.86**	0.13		**0.48**	**0.84**	0.16	
INS	**0.42**	0.39		0.37	**0.45**	**0.42**		**0.43**
GLUC†	0.31	-0.18		-0.34	-0.26	-0.24		-0.38
LDL	0.12			0.24	0.12	0.11		0.28
HDL	-0.27	-0.13		**-0.50**	-0.28	-0.14		**-0.47**
TG	0.31			**0.57**	0.28			**0.51**
SBP	0.14		**0.99**		0.15		**0.99**	
DBP		-0.15	**0.75**			-0.19	**0.76**	
WAIST	**0.70**	**0.67**			**0.73**	**0.65**		
WHR	**1.00**				**1.00**			
%BF	0.33	**0.65**			0.35	**0.63**		

Rotation="Varimax"	Blacks: All Data				Blacks: Excluding T2D			
	Factor 1	Factor 2	Factor 3	Factor 4	Factor 1	Factor 2	Factor 3	Factor 4
Proportion of Variance	**0.23**	**0.14**	**0.14**	**0.09**	**0.23**	**0.15**	**0.14**	**0.09**
Cumulative Variance	**0.23**	**0.37**	**0.51**	**0.60**	**0.23**	**0.37**	**0.51**	**0.61**
Loadings: BMI	**0.95**		0.22		**0.95**		0.23	
INS	**0.42**		**0.52**	0.15	**0.44**		**0.58**	0.15
GLUC	- 0.20		**- 0.43**	- 0.12	- 0.22		**-0.47**	
LDL			0.27				0.31	
HDL			**- 0.57**		-0.12		**- 0.54**	-0.10
TG			**0.64**	0.11			**0.57**	0.12
SBP	0.13	**0.99**			0.15	**0.99**		
DBP		**0.76**				**0.78**		
WAIST	**0.86**		0.24	**0.40**	**0.86**		0.24	**0.40**
WHR	0.32		0.31	**0.90**	0.32		0.28	**0.90**
%BF	**0.71**		0.19		**0.70**		0.20	

Rotation="None"	Whites: All Data				Whites: Excluding T2D			
	Factor 1	Factor 2	Factor 3	Factor 4	Factor 1	Factor 2	Factor 3	Factor 4
Proportion of Variance	**0.32**	**0.12**	**0.09**	**0.04**	**0.22**	**0.18**	**0.12**	**0.08**
Cumulative Variance	**0.32**	**0.44**	**0.53**	**0.57**	**0.22**	**0.39**	**0.51**	**0.59**
Loadings: BMI	**0.90**	0.14		-0.36	**0.50**	**0.82**		
INS	**0.54**	0.19	0.37	-0.15	**0.40**	**0.46**		0.35
GLUC	-0.33		-0.19		-0.23	-0.20		-0.13
LDL					0.10			
HDL	-0.25		**-0.64**		-0.23	-0.16		**-0.62**
TG	0.28	0.14	**0.58**		0.27	0.18		**0.56**
SBP	0.19	**0.86**		0.19	0.17	0.24	**0.88**	
DBP		**0.72**		0.23	0.10	0.11	**0.73**	
WAIST	**1.00**				**0.73**	**0.65**		
WHR	**0.72**		0.17	**0.40**	**1.00**			
%BF	**0.77**			-0.22	**0.45**	**0.67**	-0.11	

Rotation="Varimax"	Whites: All Data				Whites: Excluding T2D			
	Factor 1	Factor 2	Factor 3	Factor 4	Factor 1	Factor 2	Factor 3	Factor 4
Proportion of Variance	**0.25**	**0.13**	**0.13**	**0.07**	**0.25**	**0.13**	**0.12**	**0.09**
Cumulative Variance	**0.25**	**0.37**	**0.50**	**0.57**	**0.25**	**0.38**	**0.50**	**0.59**
Loadings: BMI	**0.94**	0.23			**0.93**		0.22	
INS	**0.46**	**0.51**	0.13		**0.48**	0.15	**0.49**	
GLUC	- 0.26	-0.27	**- 0.28**	- 0.10	- 0.23		- 0.20	
LDL							0.13	
HDL	-0.11	**-0.68**			- 0.12		**- 0.67**	
TG	0.11	**0.64**			0.14		**0.63**	
SBP	0.14		**0.88**		0.14	**0.92**		
DBP			**0.76**			**0.74**		
WAIST	**0.86**	0.21		**0.47**	**0.88**		0.20	0.38
WHR	**0.40**	0.27		**0.69**	0.38		0.24	**0.89**
%BF	**0.78**	0.17		0.16	**0.79**		0.16	0.12

† Variables were adjusted for age and center within ethnicity and gender (see Material and Methods)

† GLUC negative loadings are result of inverse squared power transformation of the original GLUC; † Loadings ≥ 0.4 are in bold
